# Crisis Management of Emerging/Re-Emerging Infectious Diseases in People with HIV in Japan

**DOI:** 10.31662/jmaj.2025-0433

**Published:** 2025-12-12

**Authors:** Eisuke Adachi, Hiroshi Yotsuyanagi, Tomoya Saito

**Affiliations:** 1 Department of Infectious Diseases and Applied Immunology, IMSUT Hospital of the Institute of Medical Science, The University of Tokyo, Tokyo, Japan; 2 Japan Institute for Health Security, Tokyo, Japan; 3 Center for Emergency Preparedness and Response, National Institute of Infectious Diseases, Japan Institute for Health Security, Tokyo, Japan

**Keywords:** people with HIV, emerging infectious diseases, COVID-19, mpox, health equity

## Abstract

Emerging and re-emerging infectious diseases have disproportionately affected marginalized groups such as people with HIV (PWH), yet their specific needs are often overlooked. Drawing on the experience of IMSUT Hospital in Minato ward, central Tokyo―a district that includes Roppongi and Ginza, and serves as a referral center for HIV care―during the COVID-19 pandemic, we highlight how structural factors, stigma, and delayed access to care shaped the impact on PWH. While general medical responses evolved rapidly, HIV-specific vulnerabilities such as vaccine non-responsiveness and prolonged infection persisted. The subsequent mpox outbreak in Tokyo, affecting many PWH, further illustrated how emerging infections in marginalized communities can present with population-specific complications influenced by social and epidemiological contexts. Without such measures, outbreaks among PWH and other vulnerable groups may remain undetected for longer periods and spread more widely, thereby deepening existing disparities.

## Introduction

Emerging infectious diseases disproportionately affect socially marginalized groups such as people with HIV (PWH). However, data on these populations are often scarce, resulting in delayed and inequitable responses. Globally, discussions about HIV in high-income countries tend to center on men who have sex with men (MSM). Yet, the majority of PWH live in sub-Saharan Africa, where women account for most cases ^(1)^. Despite their substantial burden, evidence regarding women living with HIV remains limited due to structural and societal barriers that restrict access to research and care. Furthermore, transgender women (TGW) have a higher HIV prevalence than MSM, but recognition of TGW as a key population has only recently gained attention ^(2)^. These disparities highlight persistent inequities in global HIV research and underscore the need to broaden perspectives beyond traditionally studied groups.

Against this background, IMSUT Hospital, affiliated with the Institute of Medical Science, The University of Tokyo, has been caring for PWH in Japan since the 1980s and was at the forefront of the COVID-19 response from the earliest stages. Located in Minato ward, Tokyo, the hospital has played a central role in both HIV care and national COVID-19 efforts. Infectious disease specialists with expertise in HIV leveraged their experience to address emerging and re-emerging infectious diseases in populations with diverse social and clinical needs.

We report here a narrative opinion based on the experiences of an HIV-specialized hospital located in central Tokyo. Drawing on retrospective clinical observations, we describe how the institution responded to the COVID-19 pandemic and the mpox outbreak among PWH, highlighting the challenges encountered and lessons learned. Through these reflections, we aim to consider approaches to crisis management for socially marginalized minority populations.

## Early COVID-19 Response in Minato Ward, Tokyo

In March 2020, Minato ward, Tokyo―located in central Tokyo and encompassing prominent districts such as Roppongi and Ginza―was among the first areas in Japan to report a COVID-19 cluster without an identifiable epidemiological link. By late March, the Governor of Tokyo publicly announced that nightlife districts were becoming the epicenter of transmission. However, institutions such as IMSUT Hospital and the Minato Public Health Center had already initiated response measures in early March. Although some cases were indeed associated with nightclubs―a setting recognized as high-risk―clusters also emerged in various occupational and social contexts. The stigmatization of certain professions subsequently arose, underscoring the importance of safeguarding the social rights of affected individuals even amidst a pandemic.

**Figure 1.**
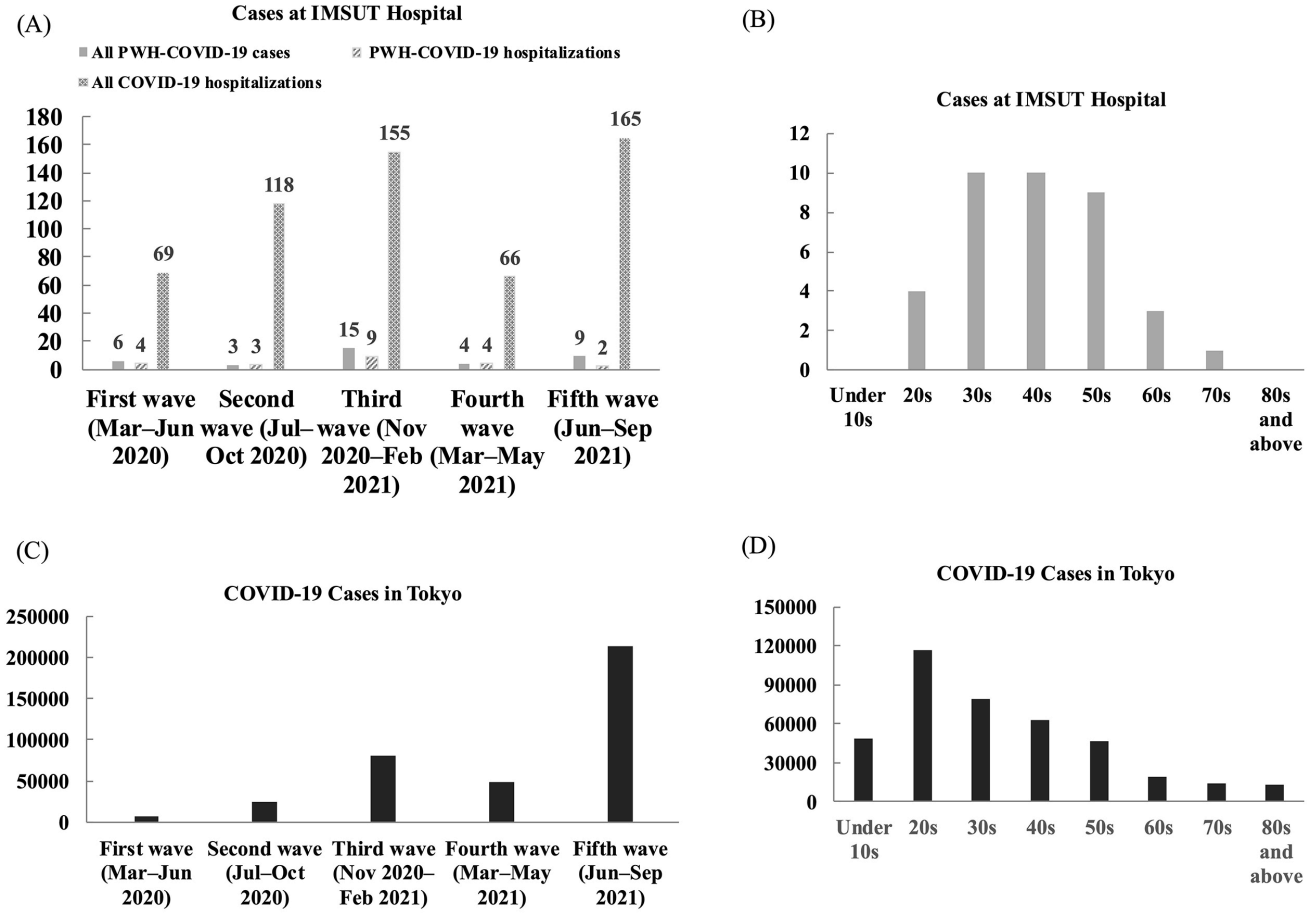
Overview of COVID-19 cases among people with HIV (PWH) at IMSUT Hospital from the first to fifth waves. IMSUT Hospital, affiliated with the Institute of Medical Science, the University of Tokyo, is a specialized center providing care for PWH. (A) Number of all PWH-COVID-19 cases and hospitalized COVID-19 cases among PWH at IMSUT Hospital during each wave. (B) Age distribution of COVID-19 cases within the PWH cohort at IMSUT Hospital. (C) Total number of COVID-19 cases in Tokyo. (D) Age distribution of COVID-19 cases in Tokyo. Data for panels C and D were calculated from data provided by the Tokyo Metropolitan Government, Bureau of Health and Medical Care, Tokyo Metropolitan Government Open Data Catalog Site (https://portal.data.metro.tokyo.lg.jp/), accessed August 1, 2025. COVID-19: coronavirus disease 2019; PWH: people with HIV.

During the first wave (March-June 2020), four people with HIV (PWH) were hospitalized for COVID-19 at IMSUT Hospital, while two others underwent isolation at home. Under Japan’s Infectious Disease Control Law, hospital admission was limited to selected cases; therefore, these four hospitalizations represented a notably high proportion―5.8% (4/69)―of all COVID-19 admissions at our institution (Figure 1A). Among these six individuals, four were MSM, and two were TGW; notably, four (two MSM and two TGW) were employed in nightclubs ^(3)^. This relatively high hospitalization rate likely reflected facilitated access to admission among PWH already engaged in specialized HIV care at our hospital.

## Impact of the COVID-19 Pandemic on Access to Care for People with HIV

In the summer of 2021, during Japan’s fifth wave of COVID-19 driven by the Delta variant, a man in his forties presented with a fever to a hospital in eastern Tokyo. Computed tomography imaging showed ground-glass opacities, raising suspicion for COVID-19, and he was transferred over 60 km to western Tokyo. During this healthcare crisis, the usual medical referral system was disrupted, and access to general hospital care for PWH in central Tokyo became severely limited. PCR testing later confirmed that he did not have COVID-19. He was eventually diagnosed with Pneumocystis pneumonia and HIV infection and admitted to IMSUT Hospital.

In contrast, PWH with COVID-19 at our institution had relatively favorable access to hospitalization through the first to fourth waves, likely reflecting the advantage of being under the care of infectious disease specialists.

## COVID-19 Incidence in People with HIV

From the first through the fifth waves, IMSUT Hospital recorded 37 COVID-19 cases among PWH (Figure 1A). Thirty-four were male, and three were female (including two TGW). Excluding the fifth wave, when vaccination had begun to affect incidence, the cumulative infection rate among our PWH cohort was 5.4% (29 out of 540 individuals), compared to approximately 1.1% (160,000 out of 14 million people) in Tokyo overall and approximately 1.7% (160,000 out of 9.3 million working-age individuals) in the working-age population―suggesting a higher infection risk among PWH (relative risk 4.7, 95% confidence interval 3.2-6.7 vs. Tokyo overall; p < 0.001; χ^2^; analysis performed using R version 4.5.1) ^(4)^.

In Tokyo overall, COVID-19 cases were most frequent among people in their 20s, with the incidence gradually decreasing in older age groups and relatively lower numbers in those over 60 (Figure 1D). In contrast, within our PWH cohort, the number of cases among individuals in their 20s was comparatively low―similar to that in older age groups―and most infections occurred in people in their 30s to 50s, which may partly reflect the age distribution of our clinic population ^(4)^. Despite fewer cases in younger PWH, the overall incidence remained high.

During the fifth wave―the largest nationwide surge before the Omicron era―the number of cases among our PWH cohort was relatively low (Figure 1A and C). This may be due to early vaccination of PWH starting in June 2021 as a high-risk group. At our institution, all people with HIV (PWH) diagnosed with COVID-19 during the fifth wave were unvaccinated.

As shown in Figure 1A, although the healthcare environment changed during each wave up to the fourth, hospitalizations of PWH at our institution remained relatively frequent. However, considering the large overall number of COVID-19 patients during the fifth wave, the proportion of hospitalized patients who were PWH was markedly lower. This finding likely reflects the higher vaccination coverage among PWH during this period.

## Clinical Characteristics of COVID-19 in People with HIV

Isolation status, disease severity, treatment, and risk factors are summarized in Table 1. Of 37 cases, 22 were hospitalized and 15 were managed at home. Under Japan’s Infectious Disease Control Law, HIV was considered a risk factor for admission. No patient required intubation. Twelve had moderate disease, some treated with high-flow oxygen, tocilizumab, or corticosteroid pulse therapy. All were on antiretroviral therapy; one was elderly, seven had a comorbidity, and three had multiple. While early cases were mostly mild, the need for oxygen rose to about 30% from the third wave, reflecting an aging cohort with more comorbidities.

**Table 1.** COVID-19 among People with HIV at IMSUT Hospital.

Total cases, n		37*
Isolation status, n (%)	Hospitalized	22 (59)
	Recuperation facility/home	15 (41)
Disease severity, n (%)	Mild	25 (68)
	Moderate	12 (32)
Treatment, n (%)	Oxygen therapy	9 (24)
	Dexamethasone and/or Baricitinib	7 (19)
	Methylprednisolone pulse and/or tocilizumab	2 (5.4)
Risk factors for severe disease, n (%)	Hypertension	8 (22)
	Diabetes mellitus	6 (16)
	Dyslipidemia	7 (19)
	Liver impairment	5 (14)
	Not on ART	2 (5.4)

ART: antiretroviral therapy. *All cases were laboratory-confirmed COVID-19; asymptomatic individuals and asymptomatic contacts identified only through screening testing are not included.

## HIV-specific Considerations in the Omicron Era

During the Omicron era, issues specific to HIV/AIDS-related cellular immunodeficiency became more apparent, including vaccine non-responsiveness and prolonged COVID-19 infection ^(5)^. Prolonged infection lasting several months can result from impaired post-infection immunity, such as inadequate antibody responses. The aforementioned Pneumocystis pneumonia case had received an mRNA COVID-19 vaccine but did not mount an antibody response. Vaccine non-responsiveness remains an unresolved research challenge for PWH, extending to other emerging infectious diseases.

## Lessons from Other Emerging Infections: Insights from the Mpox Outbreak

Outbreaks of emerging infectious diseases have significant social impacts, and responses for minority groups are often delayed and poorly documented. COVID-19 care for PWH in Japan was strongly influenced by the structure of the healthcare system ^(6)^. However, it is not uncommon for emerging infections to disproportionately affect minority communities. For example, mpox has shifted from traditional zoonotic transmission to sustained human-to-human transmission. In Europe, North America, and Japan, the current outbreaks primarily affect MSM. In contrast, in African regions, both clade I and clade II mpox still show significant transmission among children and household contacts, reflecting different epidemiological patterns. These variations parallel distinct HIV transmission dynamics globally, where heterosexual transmission predominates in Africa, while MSM populations are most affected in Japan and Western countries. In 2023, a small mpox outbreak occurred among MSM, most of whom were PWH, in Tokyo. IMSUT Hospital managed 12 such cases, some complicated by conditions specific to PWH, such as co-infection with community-associated methicillin-resistant *Staphylococcus aureu* ^(7)^. This illustrates how the clinical characteristics of emerging and re-emerging infections can be shaped by the background factors of the affected population.

## Implications

The emergence of re-emerging infections such as mpox, which initially spread among minorities, including PWH, highlights the importance of integrating minority perspectives into crisis management frameworks. As shown in this study, minorities may experience distinct patterns of healthcare access, sometimes advantageous, but often constrained by structural and social factors. Public health systems should anticipate and address the specific vulnerabilities of communities with unique social and clinical profiles by incorporating minority-inclusive strategies and maintaining equitable and timely access to care. Such approaches are critical for reducing the impact of future emerging and re-emerging infections in these populations.

## Article Information

### Acknowledgments

We sincerely thank the physicians and medical staff at IMSUT Hospital, as well as the Minato ward public health center and other healthcare institutions in Tokyo, for their dedicated support during the COVID-19 pandemic.

### Author Contributions

Conceptualized this project: Eisuke Adachi, Hiroshi Yotsuyanagi, and Tomoya Saito. Responsible for writing the manuscript and clinical management at IMSUT Hospital: Eisuke Adachi. Reviewed and approved the final manuscript: all authors

### Conflicts of Interest

None

### Ethics Approval Statement

Ethics approval was granted by the ethics board of the Institute of Medical Science, University of Tokyo (2022-48-1128). This study is an opinion paper that includes a discussion based on retrospective data. The study was approved by the institutional ethics committee and conducted using an opt-out approach with a publicly available disclosure document.

### Data Availability Statement

The data that support the findings of this study are available on request from the corresponding author. The data are not publicly available due to privacy or ethical restrictions.
